# Modeling Landscape Change Effects on Stream Temperature Using the Soil and Water Assessment Tool

**DOI:** 10.3390/w10091143

**Published:** 2018-08-27

**Authors:** Mamoon Mustafa, Brad Barnhart, Meghna Babbar-Sebens, Darren Ficklin

**Affiliations:** 1 School of Civil & Construction Engineering, Oregon State University, Corvallis, OR 97330, USA; meghna@oregonstate.edu; 2 Western Ecology Division, National Health and Ecological Effects Laboratory, Office of Research and Development, U.S. Environmental Protection Agency, Corvallis, OR 97330, USA; bradleybarnhart@gmail.com; 3 Department of Geography, Indiana University, Bloomington, IN 47405, USA; dficklin@indiana.edu

**Keywords:** stream temperature, SWAT, Marys River watershed

## Abstract

Stream temperature is one of the most important factors for regulating fish behavior and habitat. Therefore, models that seek to characterize stream temperatures, and predict their changes due to landscape and climatic changes, are extremely important. In this study, we extend a mechanistic stream temperature model within the Soil and Water Assessment Tool (SWAT) by explicitly incorporating radiative flux components to more realistically account for radiative heat exchange. The extended stream temperature model is particularly useful for simulating the impacts of landscape and land use change on stream temperatures using SWAT. The extended model is tested for the Marys River, a western tributary of the Willamette River in Oregon. The results are compared with observed stream temperatures, as well as previous model estimates (without radiative components), for different spatial locations within the Marys River watershed. The results show that the radiative stream temperature model is able to simulate increased stream temperatures in agricultural sub-basins compared with forested sub-basins, reflecting observed data. However, the effect is overestimated, and more noise is generated in the radiative model due to the inclusion of highly variable radiative forcing components. The model works at a daily time step, and further research should investigate modeling at hourly timesteps to further improve the temporal resolution of the model. In addition, other watersheds should be tested to improve and validate the model in different climates, landscapes, and land use regimes.

## 1. Introduction

Stream temperature is an important water quality parameter that affects physical and chemical processes in streams [1]. Higher stream temperatures in river systems represent a growing concern worldwide and can affect the habitat and life spans of fish [2,3]. According to Eaton and Scheller [4], some fish species will disappear from the water body, if stream temperature transcends an upper limit. Using historical data ranging from 30 to 100 years, Kaushal et al. [5] reported that stream temperatures have been increasing throughout the United States at a rate of 0.009–0.077 _C/year, with a significant increase in the western United States. In particular, stream temperatures in the Pacific Northwest have reached historical records—at times, they have exceeded the lethal limit of 21.1 _C for some aquatic species such as salmon. For example, in the summer of 2015, the river temperature in the Columbia River in the State of Washington reached the level of 24.5 °C and led to the death of 235,000 sockeye salmon out of the total 507,000 that passed through the Bonneville Dam [6].

Similarly, Marys River (Hydrologic Unit Code 17090003) is a tributary of the Willamette River in Northwest Oregon and has experienced increasing temperatures over the last few years. The OregonDepartment of Fish and Wildlife (ODFW) conducted a stream survey on flow conditions between the years of 1991 and 1993 and observed that the maximum stream temperature reached the maximum limit of 17.8 °C (ODFW). However, recently, increasing levels of human activities have resulted in even higher water temperatures. For example, high water temperatures between 21.1 °C and 26.7 °C were observed from June to August for some tributaries of Marys River (Marys River Watershed Council), which has resulted in several United States Environmental Protection Agency (USEPA) 303(d) listings for temperature exceedances. These trends may be related to changing climatic drivers as well as land use practices (e.g., harvest of timber, increasing barren lands and clear-cutting areas throughout the watershed) and landscape changes (e.g., urban and agricultural development).

Amidst large-scale landscape and land use changes, preservation of riparian buffers can increase stream shading, thereby helping regulate water temperature along stream reaches [7]. Stream shading intercepts and absorbs a large portion of solar radiation before it reaches the water surface, resulting in less thermal energy that reaches and is stored in streams, which indirectly helps to cool stream temperatures. Brown et al. [8] conducted a study in the Alsea watershed in Oregon along the coast range to study the impact of shading on stream temperatures before and after clear cuts in the watershed. They found that clear-cutting resulted in stream temperature increases of 7.8 ^◦^C one year after the cuts. Bond et al. [9] investigated the impact of riparian reforestation on summer stream temperatures in the Salmon River in northern California, and they found that partial reforestation lowered stream temperatures by 0.11–0.12 °C/km and by 0.26–0.27 °C/km for full reforestation.

Since increasing water temperatures have remained a major concern in many watersheds, many models have been proposed to simulate stream temperatures at time scales varying from minutes to months (see Ficklin et al. [10] for a brief review). For this paper, we specifically focus on a semi-distributed mechanistic watershed model called the Soil and Water Assessment Tool (SWAT) [11], which has been extensively used to evaluate the effects of landscape and land use changes on different hydrologic components. Ficklin et al. [10] improved the original stream temperature model within SWAT, which was a linear regression model by Stefan and Preud’homme [12] that correlates thermal energy exchange of air temperature to water temperature. Ficklin et al. [10] developed a daily-scale model for stream temperature prediction by integrating multiple climate and hydrological components, including snowmelt, surface runoff, lateral flow, groundwater flow, and finally air temperature. Several studies have found the Ficklin et al. model [10] produces more realistic simulation results compared to the linear regression proposed by Stefan and Preud’homme [12] (Barnhart et al., 2014; Ficklin et al., 2012; Ficklin et al., 2014 [10,13,14]). However, the model developed by Ficklin et al. [10] does not explicitly account for the different types of radiation that affect thermal energy of water systems, and only recent work has attempted to improve the model by incorporating select radiative components [15]. In general, thermal energy added and removed from any water system consists of incoming radiation that adds thermal energy to the water and results in increasing stream temperatures. This incoming radiation mainly consists of solar radiation coming from the sun, atmospheric longwave radiation, landcover longwave radiation, convection, and evaporation. In contrast, backscattered radiation removes thermal energy and helps to cool temperatures. This radiation consists of emitted longwave radiation from the water surface as well as convection and evaporation. Convection and evaporation radiative components can either add or remove energy from any water body depending on stream temperatures and climate conditions, specifically air temperature, humidity, and wind speed.

This paper highlights model development to explicitly incorporate multiple thermal radiation components into the Ficklin et al. [10] stream temperature model within SWAT. These thermal radiation equations are used within the widely used HEATSOURCE model [16], but until now, these equations have yet to be incorporated into SWAT. HEATSOURCE differs from SWAT because it is a reach-based stream temperature model, whereas SWAT is a watershed model. This means that SWAT simulates upland processes in addition to in-stream processes. HEATSOURCE can model stream temperatures at hourly timesteps and requires site-specific data (e.g., shading, canopy structure, stream morphology) that is oftentimes not available over the entire spatial extent of watersheds. Conversely, SWAT simulates hydrologic components and stream temperatures throughout a watershed using a daily time step and utilizes generally obtainable input data, such as spatially distributed precipitation, temperature, elevation, land use, and soil type. Users may prefer to use SWAT instead of HEATSOURCE when site-specific data is unavailable or when the study goal is to determine the effect of alternative land management scenarios on stream temperatures throughout large, heterogeneous watersheds.

The paper is organized as follows. First, the study area and the SWAT model setup are described. Then, three different SWAT stream temperature models are analyzed, including Stefan and Preud’homm [12] air temperature regression, a mechanistic model by Ficklin et al. [10], and an extension of the Ficklin et al. [10] model in which we specifically incorporate radiative components. We calibrate SWAT for hydrologic discharge in the Marys River watershed, and we compare the three stream temperature models to examine their relative performance for multiple sub-basins with different land use/land cover. We demonstrate the utility of our results by comparing simulations for sub-basins within primarily forested and agricultural landscapes.

## 2. Methodology

### 2.1. Study Area

**Figure 1.**
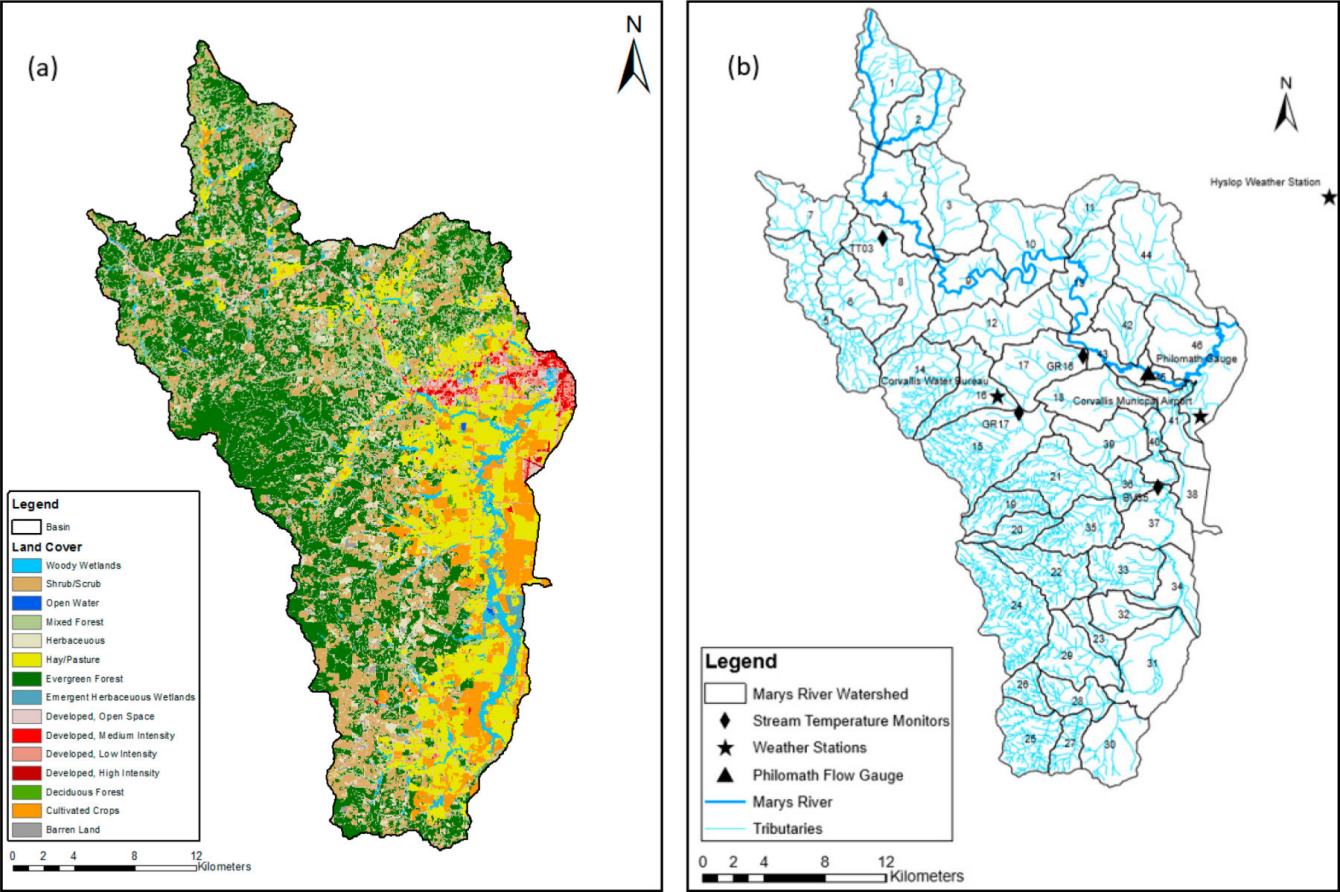
(**a**) Overview of Marys river watershed, and (**b**) land use in Oregon, USA.

The Marys River watershed, shown in Figure 1, is located in the Pacific Northwest of the United States (Hydrologic Unit Code (HUC) 17090003) and is part of the Willamette River basin (HUC 170900) in Oregon. It is one of five major river systems located on the western side of the Willamette River. The area of the watershed is of 782 square kilometers. The highest point of the watershed is at Marys Peak at an elevation of 1280 meters above sea level, and the lowest point is in Corvallis, Oregon, where Marys River drains into the Willamette River at an elevation of 76 meters above sea level. The climate of the watershed in the winter season is mild and wet, with an average winter temperature of 5 °C and rainfall during the winter ranges from 1000 mm downstream of the watershed to more than 2500 mm at the highest elevation upstream of the watershed. In general, the watershed tends to be dry, sunny, and warm throughout the summer (Marys River Watershed Council). It has an average summer temperature of 17.5 °C. High rainfall intensity results in high stream discharge during winter and spring and mean annual flows of 12–13 m^3^/s. However, during the summer, flows are generally very low, and discharge sometimes drops below one cubic meter per second. Base flow is a major contributor to the flow of the river, where 61–70% of the total stream flow comes from groundwater contributions [17].

The watershed is divided into three different land use categories: Forest, agricultural, and urban. Most of the watershed (65% of the total area) consists of forest, which is largely located along the western portion of the watershed. In these mixtures of deciduous and evergreen forests, small streams flow over beds of gravel and cobbles with high velocities due to steep slope gradients. Flow leaving the forested region then enters agricultural land in the Willamette Valley that consists mainly of cultivated crops, hay, pasture, wheat, and grass seed production. The streams in this region flow on sand and silt with mild slope gradients, resulting in decreased flow velocities. Furthermore, urban areas are situated further downstream within the Willamette Valley (e.g., the cities of Philomath and Corvallis), and the stream flows over mostly flat to nearly flat gradients. Stream velocities decrease significantly as Marys River enters Philomath, Oregon, and then continues eastwards into Corvallis, Oregon, until it meets the Willamette River at the lowest point located in the watershed.

### 2.2. Soil and Water Assessment Tool (SWAT)

The Soil and Water Assessment Tool (SWAT) is a semi-distributed watershed model that is designed to predict the impact of management on water, sediment, and agricultural chemical yields in gauged and ungauged watersheds [11]. In this study, SWAT was used to simulate the hydrologic and stream temperature dynamics within the Marys River watershed and to evaluate a model extension to the stream temperature model developed by Ficklin et al. [10]. The Marys River watershed was divided into smaller sub-watersheds using pre-defined drainage boundaries and a 10-meter digital elevation model, and then the sub-watersheds in the SWAT model were further divided into smaller units called hydrologic response units (HRUs) using ArcSWAT, a toolbox within ArcGIS for SWAT, with a HRU percentage threshold of 5%. Each HRU is a unique combination of land-use, soil type, and topographic slope and represents the basic unit for conducting mass balances and hydrologic flow in SWAT. The area of the 46 predefined sub-basins varies from 35 square kilometers for the largest sub-basin to 3.0 square kilometers for the smallest. The average sub-basin area is 17 square kilometers. The watershed slope was divided into two categories: (1) A steep gradient area located mostly within the forested regions in the western portion of the watershed, and (2) the nearly flat region located within the Willamette valley, east of the watershed where the cities Philomath and Corvallis are located.

SWAT’s input data types include spatial GIS input files such as a Digital Elevation Model (DEM), a land use land cover layer, and a soil layer [18]. Input data needed to delineate the watershed including the DEM, sub-basins, and stream layers in addition to necessary land use and soil SSURGO (Soil Survey Geographic Database) layers to build the HRUs were acquired from United States Department of Agriculture [19]. Three weather stations were used as climate forcings: Corvallis Water Bureau (CWB) COOP ID of (351877), Hyslop weather station, which is also known as Oregon State University weather station (OSU) COOP ID of (351862), and finally Corvallis municipal airport (KCVO) weather station. Weather data of the Corvallis Water Bureau (CWB) and Hyslop weather station were obtained from National Oceanic and Atmospheric Administration (NOAA) for 2005 to 2014. Weather data for Corvallis Water Bureau station included only precipitation and minimum and maximum air temperature. The Hyslop weather station and Corvallis municipal airport stations included data for precipitation, minimum and maximum temperature, wind speed, and humidity for the period of 2005 to 2014. SWAT was used to simulate flow and stream temperature throughout the Marys River watershed for the period 2005–2014. This includes the period 2010–2014 when observations for stream temperature were available.

### 2.3. Stream Temperature Models

#### 2.3.1. Model 1: Linear Regression

The default SWAT stream temperature model uses a linear relation between air temperature and stream temperature developed by Stefan and Preud’homme [12] to calculate stream temperature in the Mississippi River basin, as shown in Equation (1):

(1) $${\text{T}}_{\text{water}}=5.0+0.75\times {\text{T}}_{\text{air}}$$

T_water_ is the average daily water temperature (°C), and T_water_ is the average daily water temperature (°C). Stream temperatures predicted from the above equation will always be higher than air temperature, which is generally a fair assumption for small streams with shallow water depths where stream temperature is primarily controlled by air temperature. However, this may not be necessarily true for streams influenced by snowmelt, surface runoff, and groundwater contributions [10].

#### 2.3.2. Model 2: A Mechanistic Approach Involving Air Temperature and Hydrological Flows

Ficklin et al. [10] developed a mechanistic stream temperature model within SWAT by combining air temperature (heat exchange) and hydrological inputs (flow mixing) including different hydrological parameters, surface runoff, lateral flow, snowmelt, and groundwater contributions. The Ficklin et al. [10] stream temperature model discretizes stream temperature determination into three components: (1) Within the sub-basin, (2) contribution of upstream sub-basins to the targeted sub-basin, and (3) finally heat exchange between air temperature and the stream.

The first part of the stream temperature calculation within the sub-basin (Equation (2)) calculates the local temperature based on a mixing of surface runoff, lateral flow, groundwater, and snowmelt temperatures within the sub-basin flowing to the main stream:

(2) $${\text{T}}_{\text{w},\text{local}}=\frac{\alpha \left(0.1\;{\text{Sub}}_{\text{snow}}\right)+\beta \left({\text{T}}_{\text{gw}}\;{\text{Sub}}_{\text{gw}}\right)+\lambda \left({\text{T}}_{\text{air},\;\text{lag}}\;{\text{Sub}}_{\text{surq}}+{\text{Sub}}_{\text{latq}}\right)}{{\text{Sub}}_{\text{w}}{}_{\text{yld}}}$$

T_air,lag_ is average daily air temperature with a lag (°C), and α, β, and λ are calibration coefficients that relate the relative contribution of the hydrologic components to local water temperature (dimensionless). Sub_snow_ is the snowmelt contribution in sub-basin (m^3^/day), Sub_gw_ is the groundwater contribution in sub-basin (m^3^/day), Sub_surq_ is the surface runoff in the sub-basin (m^3^/day), Sub_latq_ is the lateral soil flow in sub-basin (m^3^/day), and Sub_wyld_ is the water yield in the sub-basin combining all of the above hydrological inputs (m^3^/day).

The second part of the Ficklin et al. [10] calculates the effect of upstream sub-basin flow on stream temperature, as shown in Equation (3):

(3) $${\text{T}}_{\text{waterinitial}}=\frac{({\text{T}}_{\text{w},\text{upstream}})({\text{Q}}_{\text{outlet}}{\text{-Sub}}_{\text{wyld}})+\left({\text{T}}_{\text{w},\text{local}}\times {\text{Sub}}_{\text{wyld}}\right)}{{\text{Q}}_{\text{outlet}}}$$

T_waterinitial_ is the stream temperature adding the effects of flow within the sub-basin, T_w,local_ was calculated previously, T_w,upstream_ is the water temperature of streams entering the sub-basin (°C), and Q_outlet_ is the stream flow discharge at the outlet of sub-basin (m^3^/day).

The final step is to calculate the stream temperature by including the effect of air temperature:

(4) $${\text{T}}_{\text{water}}={\text{T}}_{\text{waterinitial}}+\text{K}\left({\text{T}}_{\text{air}}-{\text{T}}_{\text{waterinitial}}\right)(\text{TT})\;\text{if}\;{\text{T}}_{\text{air}}>\text{0}\;{\text{T}}_{\text{water}}={\text{T}}_{\text{waterinitial}}+\text{K}(\left({\text{T}}_{\text{air}}+\varepsilon \right)-{\text{T}}_{\text{waterinitial}}(\text{TT})\;\text{if}\;{\text{T}}_{\text{air}}<\text{0}$$

T_water_ is the final stream temperature of water (°C) for a given sub-basin, T_air_ is the average daily air temperature (°C), K is the bulk coefficient of heat transfer (1/h), TT is travel time of water through the sub-basin (hour), and finally ε is air temperature addition coefficient (for when air temperature drops below zero).

This mechanistic stream temperature model requires calibration coefficients α, β, γ, k, lag as well as annual groundwater temperatures as inputs. All of the other inputs needed to run the model are provided by SWAT. Groundwater temperature can be estimated from weather data provided as the annual average air temperature, and it is often taken 1–2 °C higher than the average annual air temperature [20].

#### 2.3.3. Model 3: A Mechanistic Approach Involving Air Temperature, Hydrological Flows, and Radiative Components

Changes in stream temperature are affected by heat and mass transfers [16] that are dependent on channel morphology, hydrology, and stream vegetation, which provides shading near streams. Vegetation especially helps in cooling temperatures by intercepting and absorbing incoming solar radiation. The mechanistic model introduced in the last section accounts for flow transfer and mixing of various hydrologic components, including sub-basin surface runoff, lateral flow, snowmelt, and ground water, which oftentimes help to reduce temperatures, depending on the season. However, the model utilizes a bulk heat coefficient to account for radiative heat exchange between the air-water interface and does not account for land cover or vegetation near streams. The dependency of the model on the air-water correlation of heat exchange can thereby lead to over-prediction of stream temperatures. Water temperature change related to heat transfer is a function of several sources of radiative heat exchange, as shown in Equation (5):

(5) $$\Phi_{\text{total}}=\Phi_{\text{SR}}+\Phi_{\text{longwave-atmosphere}}+\Phi_{\text{longwave-landcover}}+\Phi_{\text{convection}}+\Phi_{\text{conduction}}+\Phi_{\text{evaporation}}$$

where, Φ_total_ is the net radiation exchange and is equal to the direct and diffuse solar radiation Φ_SR_ as well as the longwave-atmosphere, longwave-landcover, convection, and evaporation components.

Direct and diffusive solar radiation represent the largest sources of incoming thermal energy into streams. Longwave radiation from different sources also plays a role in increasing and decreasing temperatures: Atmospheric and land-cover longwave radiation add energy to water volumes and increase water temperature, while longwave radiation from the water surface emits radiation from the water surface to the atmosphere to cool streams. Energy loss due to evaporation is considered a larger contributor of decreasing stream temperatures when the required energy is met to change the water phase from liquid to gas. Overall, convection or the air-water interface is considered a very small portion of the total energy budget. Groundwater flux also helps to decrease stream temperatures when added as a thermal input.

These radiative components have been included in the HEATSOURCE model but have not been incorporated explicitly within spatially distributed watershed models such as SWAT. Each of the components are defined as follows.

(6) $$\Phi_{\text{SR}}={\text{H}}_{\text{day}}-0.5\times {\text{H}}_{\text{day}}\left(1-{\text{e}}^{-\text{k}\times \text{LAI}}\right)$$

Φ_SR_ is the amount of solar radiation reaching the water surface, H_day_ is the incident total solar radiation per day (MJ/m^2^·day), k is the light extinction coefficient, and LAI is the leaf area index. Atmospheric solar radiation is calculated as follows:

(7) $$\Phi_{\text{longwave-atmosphere}}=0.96\times \varepsilon_{\text{atm}}\times \sigma \times {\left({\text{T}}_{\text{air}}+273.15\right)}^{4}$$

Φ_longwave-atmosphere_ is the longwave radiation emitted from the atmosphere, ε_atm_ is the emissivity of the atmosphere (unitless), σ is the Stefan-Boltzmann constant (MJ·m^−2^·day^−1^·K^−4^), and T_air_ is the average air temperature per day (^◦^C). The atmospheric solar radiation depends on the emissivity of the atmosphere (ε_atm_), in which the percent of cloudiness and type of landuse can increase or decrease atmospheric emissivity. The emissivity of the atmosphere is calculated as $\varepsilon_{\text{atm}}=0.767\times {\left({\text{e}}_{\text{a}}\right)}^{\frac{1}{7}}$, where ea is the vapor pressure of air (mbar) (i.e., H × e_s_), e_s_ is the saturation vapor pressure (mbar) (i.e., 6.1275 × ${\text{e}}^{\frac{17.27\times {\text{T}}_{\text{air}}}{{}^{237.3+{\text{T}}_{\text{air}}}}}$), and H is the relative humidity (unitless).

The longwave radiation emitted from landcover Φ_longwave-landcover_ is dependent on the view to the sky θ_VTS_ (unitless) and can be calculated as follows:

(8) $$\Phi_{\text{longwave-landcover}}=0.96\times \left(1-\theta_{\text{VTS}}\right)\times 0.96\times \sigma \times {\left({\text{T}}_{\text{air}}+273.15\right)}^{4}$$

The last component of longwave solar radiation is the water surface longwave:

(9) $$\Phi_{\text{longwave-water}\;\text{surface}}=\varepsilon_{\text{W}}\times \sigma \times {\left({\text{T}}_{\text{s}}+273.15\right)}^{4}$$

Φ_longwave-water surface_ surface is the longwave radiation emitted from the water surface, ε_w_ is the water emissivity taken as 0.97, and T_s_ is the stream temperature (°C). The evaporation from the water surface is the most effective component in decreasing the thermal energy stored in water:

(10) $$\Phi_{\text{evaporation}}=\rho \times {\text{L}}_{\text{e}}\times \text{E}$$

ρ is the density of water (kg/m^3^), L_e_ is the latent heat of vaporization (MJ/kg), which is calculated as L_e_ = 2.501 − 2.361 × 10^−3^ × T_air_. E is the evaporation rate of the water surface (m/day) and is calculated using a mass transfer method: f(w) × (e_sw_ − e_aw_), where f(w) is the wind function a + b × w that depends on coefficients a and b (mbar^−1^) and the wind speed w (m/s) measured 2 m above the water surface [16]. Finally, e_sw_ is the saturation vapor pressure of water (mbar), and e_aw_ is the vapor pressure of water (mbar).

The convection radiation component is calculated using the previously calculated evaporative flux Φ_evaporation_ and Bowen’s ratio BR:

(11) $$\Phi_{\text{convection}}=\text{BR}\times \Phi_{\text{evaporation}}$$

Here, BR is unitless (i.e., 0.00061 × P_A_ × $\frac{{\text{T}}_{\text{water}}-{\text{T}}_{\text{air}}}{\left({\text{e}}_{\text{sw}}-{\text{e}}_{\text{aw}}\right)})$, and P_A_ is the adiabatic air pressure (mbar) [i.e., 1013 − 0.1055 × z]. z is the measurement height in meters [i.e., >zd + zo]; zd is the zero-plane displacement (m) [i.e., 0.7 × H_Lc_], zo is the roughness height = 0.1 × H_Lc_, and H_Lc_ is the height of emergent vegetation (m).

The change of stream temperature due to thermal energy flux is calculated as follows:

(12) $${\text{T}}_{\text{w}-\text{TD}}=\frac{\Phi_{\text{total}}}{\rho_{\text{w}}\times {\text{C}}_{\text{w}}\times {\text{d}}_{\text{W}}}$$

Here ${\text{T}}_{\text{w}-\text{TD}}\frac{{}^{\circ}\text{C}}{\text{day}}$ is the temperature change generated from the thermal components, Φ_total_ is Here, T_w−TD_ the net driver (MJ/m^2^·day), C_w_ (MJ/kg·C) is the specific heat capacity of water, and d_w_ (m) is the depth of water in the channel, which is estimated by the SWAT model.

The final stream temperature is calculated by replacing the second term in Equation (4) by the new generated stream temperature, as follows:

(13) $${\text{T}}_{\text{w}}{}_{\text{ater}}{=\text{T}}_{\text{waterinitial}}+{\text{T}}_{\text{w}-\text{TD}}$$

Note that, as mentioned above, the majority of the components are calculated within the SWAT model automatically.

Overall, this model is useful because it utilizes all of the distributed information (mechanistically simulated via SWAT) regarding stream temperature and its relation to hydrologic components within a networked watershed, yet it also explicitly incorporates radiative energy exchange at the surface of the stream.

### 2.4. Model Calibration/Validation Methodology

SWAT was used to simulate daily hydrologic discharge at each of the sub-basins within the Marys River watershed from 2010–2014 in order to match observed discharge and stream temperature data. The SWAT model was manually calibrated for stream flow between 2010–2014 using the United StatesGeological Survey (USGS) (14171000) Philomath flow gauge, which is located 6.7 km southwest of where the Marys River meets the Willamette River, covering a 394 km^2^.

The model was only calibrated without validation due to the availability of observations for flow since the available observations only included a period of less than 10 years. Based on the data availability, the model was calibrated for the period of January 2010 to December 2014.

The Nash Sutcliffe efficiency (NSE; Nash and Sutcliffe (1970)) criterion Equation (14) and Pearson’s product moment correlation coefficient (1999; Equation (15)) were used to evaluate hydrologic model efficiency:

(14) $$\text{NSE}=1-\frac{\sum_{\text{i}=1}^{\text{n}}{\left({\text{O}}_{\text{i}}-{\text{S}}_{\text{i}}\right)}^{2}}{\sum_{\text{i}=1}^{\text{n}}{\left({\text{O}}_{\text{i}}-{\text{O}}_{\text{avg}}\right)}^{2}}$$

(15) $${\text{R}}^{2}={\left(\frac{\sum_{\text{i}=1}^{\text{n}}\left({\text{S}}_{\text{i}}-{\text{S}}_{\text{avg}}\right)\left({\text{O}}_{\text{i}}-{\text{O}}_{\text{avg}}\right)}{{\left[{\sum_{\text{i}=1}^{\text{n}}\left({\text{S}}_{\text{i}}-{\text{S}}_{\text{avg}}\right)}^{2}\right]}^{0.5}\left[\sum_{\text{i}=1}^{\text{n}}{\left({\text{O}}_{\text{i}}-{\text{O}}_{\text{avg}}\right)}^{2}\right]}\right)}^{2}$$

O is the observed value, S is the model prediction, O_avg_ is the overall observed mean, and S_avg_ is the overall simulated mean. The NSE values range from −∞ to one; a NSE value of less than 0.5 designates an ‘unsatisfactory’ model, while a NSE value above 0.75 is considered a ‘very good’ model [21]. R^2^ values range from zero to one, with zero indicating a nonlinear relationship between the observed and predicted value and one indicating a perfect fit and a linear relationship between the observed and simulated variables.

The stream temperature models were manually calibrated using root mean square error (RMSE) values as well as percent bias (PBIAS). According to Chai et al. [22], RMSE is widely used as a statistical metric tool to assess performance of models. RMSE can be calculated as follows:

(16) $$\text{RMSE}=\sqrt{\frac{\sum_{i=1}^{n}{\left(O_{i}-S_{i}\right)}^{2}}{n}}$$

Where O is observed value, S is model predicted value, and n is the total number of the points. A RMSE value of zero indicates a perfect fit. According to Singh et al. [23], values of RMSE less than half of the standard deviation of the measured data can be taken as acceptable for the model evaluation.

In addition to RMSE, PBIAS was also used to evaluate the models. PBIAS measures the average tendency of the simulated data to be larger or smaller than their observed counterparts [24]. Zero is the optimal value of PBIAS, and a low absolute value implies an accurate model. According to Gupta et al. [24], positive PBIAS values indicate a model underestimation bias, and negative PBIAS values indicate a model overestimation bias. PBIAS can be calculated as follows:

(17) $$\text{PBIAS}=\frac{\sum_{\text{i}=1}^{\text{n}}\left({\text{O}}_{\text{i}}-{\text{S}}_{\text{i}}\right)\times 100}{\sum_{\text{i}=1}^{\text{n}}\left({\text{O}}_{\text{i}}\right)}$$

A set of seven calibration parameters were selected and manually modified to calibrate SWAT for hydrology (Table 1), and five parameters were manually modified to calibrate SWAT for stream temperature (Table 2). Observed daily stream temperature data corresponding to SWAT’s sub-basins 8, 15, and 17 were available from 2010 through 2014, while observations for sub-basin 36 extended from 2011 through 2014.

To compare the differences in model performance, kernel density estimates were calculated using R software. This nonparametric technique is similar to using histograms to highlight the differences between model simulations and observed data for the three tested models.

## 3. Results and Discussion

### 3.1. Hydrology Calibration in SWAT

**Figure 2.**
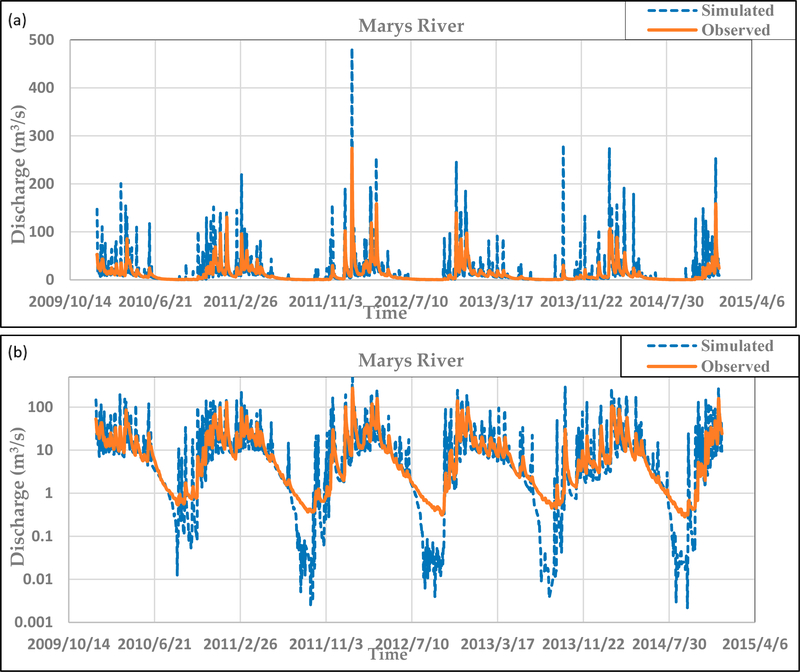
(**a**) Uncalibrated daily SWAT discharge simulations compared with observations (2010–2014) from the USGS (14171000) Philomath flow gage. (**b**) Uses a log scale to emphasize low-flow conditions.

As mentioned previously, SWAT was used to simulate daily hydrologic discharge at each of the sub-basins within the Marys River watershed for 2005–2014, which included 2010–2014, when observations for stream temperature were also available. The NSE and R^2^ values of the default model’s simulated flow (no calibration) were −0.37 and 0.50, respectively, indicating an unsatisfactory model. From Figure 2, it is clear that the uncalibrated model overpredicts the peaks during storm events. Also, the model’s responses to each rain event are very rapid, and the water loss rates are excessive, which results in zero flow for late summer periods.

**Figure 3.**
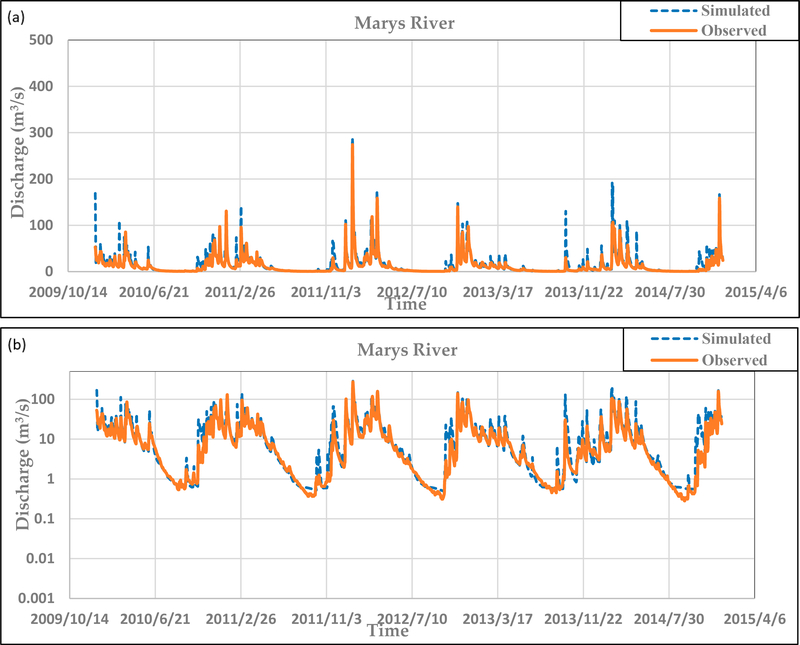
(**a**) Manually calibrated daily Soil and Watershed Assessment Tool (SWAT) discharge simulations compared with observations (2010–2014) from the United State Geological Survey (USGS) (14171000) Philomath flow gage. (**b**) Uses a log scale to emphasize low-flow conditions.

Manual calibration resulted in model improvement of NSE values from −0.37 to 0.72. This designates a ‘good’ model according to Moriasi et al. [21], since it is >0.65. The R^2^ value of the model increased to 0.80. Figure 3 shows that the calibrated model matches both the base flow and peaks well, whereas all of the peaks were mainly over predicted by the default model. The default model could not capture the very low stream flows for some summer days and resulted in zero flow (Figure 2), but the calibrated model (Figure 3) fixed the low-flow problem and improved the results. Figure 2 shows that the maximum simulated peak for the uncalibrated model was around 500 m^3^/s, whereas the calibrated model (Figure 3) reduced this value to match the peak observed around 300 m^3^/s. The rapid response of the main channel to any storm event led to the over-prediction of peaks even for small rain storms in the uncalibrated model. Also, the lateral flow travel time parameter helped to slow the response to storm events and smoothed the hydrograph. The other major problem was associated with the excessive loss of water in a short period of time after a rapid response to any storm; water in the uncalibrated model was lost instantaneously and therefore resulted in zero flow for late summer days. The effective hydraulic conductivity in the main channel (CH_K2) was used to prevent the excessive loss from the main channel and managed to eliminate the zero flow days. Also, this parameter helped to smooth and eliminate the transient fluctuations in the hydrograph. The SCS curve number for moisture conditions II (CN2) was used to reduce or increase the simulated peaks to match the observed hydrograph. The canopy interception parameter for specified land cover types (CANMX) as well as the minimum and maximum snowmelt factors (SMFMN, SMFMX) all helped to increase the evaporation rate. The high surface runoff surge was decreased using the soil evaporation compensation factor (ESCO), the SCS curve number for moisture conditions II (CN2), and the threshold depth of water in the shallow aquifer (GWQMN).

### 3.2. Stream Temperature Calibration in SWAT

After a satisfactory hydrologic calibration was performed, manual calibration was performed for two of the three stream temperature models that will be tested in the Marys River using SWAT. The first model is the linear regression model from Stefan and Preud’homme [12] and was not calibrated. The second model is the Ficklin et al. [10] model, and the third model is our extension to the Ficklin et al. [10] model. As shown in Table 2, we manually calibrated five parameters to best match the second and third models to the stream temperature observations between 2010 and 2014. Note that only summer stream temperature observations were available and that only a single set of calibration parameters were used for the entire watershed.

### 3.3. Stream Temperature Model Comparison

After satisfactory hydrologic and stream temperature calibrations were performed, three models were tested to simulate stream temperature in the Marys River using SWAT. The first was the linear regression model from Stefan and Preud’homme [12], the second the Ficklin et al. [10] model, and the third is our extension to the Ficklin et al. [10] model that incorporates HEATSOURCE radiative forcing components to the Ficklin et al. [10] model. For the remainder of this paper, these models will be referred to as Model 1, Model 2, and Model 3, respectively.

**Figure 4.**
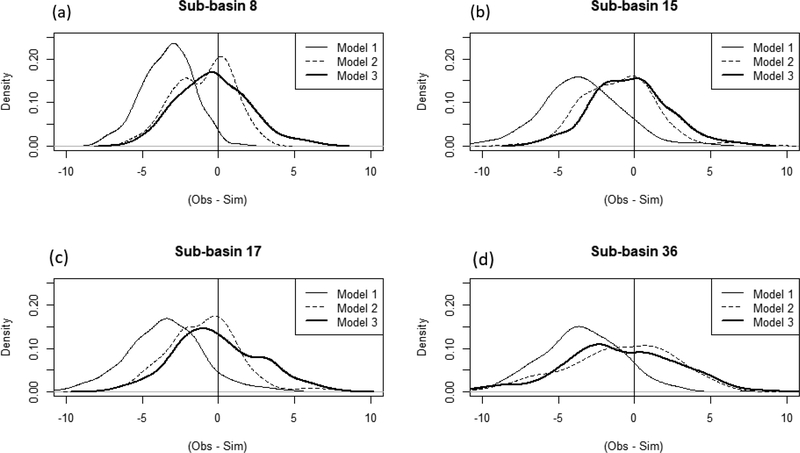
Kernel density estimates for the differences between observed and simulated stream temperatures as calculated using the three models for four sub-basins (**a**–**d**). The vertical line at zero indicates simulations that exactly match the observed data.

Table 3 shows RMSE and PBIAS results for these three models using daily data. Kernel density estimates for the differences between simulated and observed stream temperatures for the four sub-basins are plotted in Figure 4.

In general, Model 1 performed the worst among all three models (see Table 3 and Figure 4), and Model 2 outperformed Models 1 and 3 for all sub-basins. Model 1 consistently overestimated stream temperatures for all of the sub-basins. This is apparent in Figure 4, where the distributions of the differences between the simulated and observed temperatures are shifted from zero for Model 1. The coefficients of Model 1 guarantee that the simulated stream temperature will be above average daily air temperature values when the average air temperature is less than 20 ^◦^C. Therefore, inclusion of cold water from groundwater or upstream sources is not captured in this model, thus resulting in overestimations. Model 2, which is the calibrated Ficklin et al. [10] model, showed improvements compared to the linear model (Model 1) in both Table 3 and Figure 4, which agrees with previous studies (Barnhart et al., 2014; Ficklin et al., 2012; Ficklin et al., 2014) [10,13,14]. This is presumably because Model 2 incorporates hydrologic components, including groundwater upstream temperatures, in addition to an air-heat exchange transfer coefficient. Model 3, which replaced the simple air-heat exchange transfer coefficient from Model 2 with explicitly calculated radiative components, shows similar distributions with Model 2 (Figure 4), yet the performance values of Model 2 are generally better. This is likely due to the high variability of the radiative components included in Model 3, which will be discussed further in the next section.

### 3.4. Land Cover Effects on Stream Temperature

**Figure 5.**
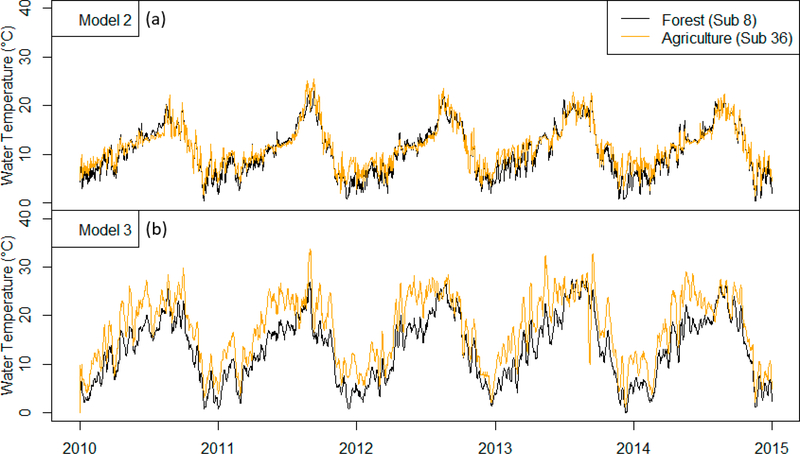
Stream temperatures simulated using the Ficklin et al. [10] model (Model 2; panel (**a**)) and the extended version that includes radiative components (Model 3; panel (**b**)). Model 2 does not capture changes in stream temperature associated with land cover type, while Model 3 simulates increased temperatures for agricultural regions.

We now compare Models 2 and 3 to demonstrate that the incorporation of radiative components is able to simulate the influence of land use and land cover on stream temperatures (e.g., forested vs. agricultural regions). Stream temperature simulations for two sub-basins—a forested area (sub-basin 8) and an area dominated by agriculture with low vegetation (sub-basin 36)—are shown in Figure 5.

**Figure 6.**
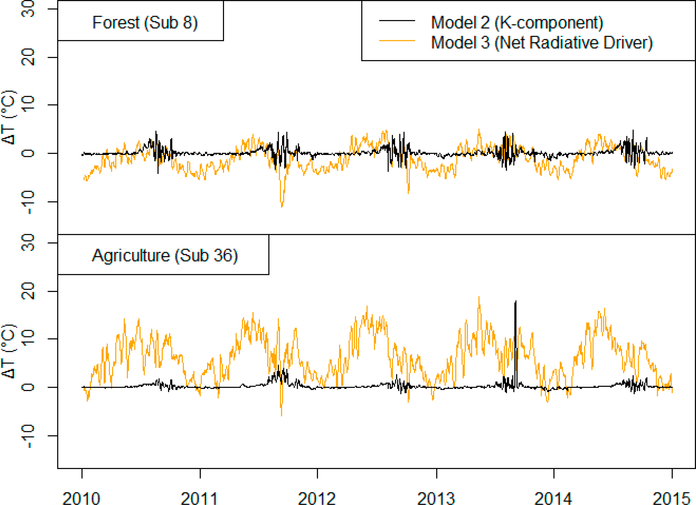
Comparison of the thermal energy impacts on stream temperatures for Models 2 and 3 in both primarily forested and agricultural sub-basins.

Model 2 simulates nearly identical stream temperatures for both forested and agricultural sub-basins. Conversely, by including the various radiative components into the model, Model 3 simulates consistently increased stream temperatures associated with the agricultural sub-basin. This is especially apparent during the early summer months. To examine differences in the models further, Figure 6 compares the net radiative components of Model 3 Equation (5) with the K-component second term in Equation (4) of Model 2.

Figure 6 shows that the net radiation as calculated using Model 3 (orange lines) changes according to the primary land use cover for a given sub-basin in SWAT. For example, agricultural sub-basins have larger incoming (positive) radiation contributions that help to increase stream temperatures, whereas forested areas have small or negative radiative effects, depending on the season, due to increased LAI, reduced solar radiation reaching the water surface, and evaporative fluxes. Model 2 uses a bulk coefficient of heat transfer in the second term of Equation (4). This reflects a convection component of the net radiative balance, but it does account for the other radiative energy terms, including solar radiation, atmospheric longwave, land surface longwave, water surface longwave, and evaporation. Therefore, it is not able to capture cover-related differences in net radiation and therefore changes in stream temperature due to landscape and land use changes.

Figure 6 also shows that the net radiative driver as calculated in Model 3 has much higher variability than the K-component used in Model 2. We found that the high variability (i.e., noise) is mainly due to SWAT’s estimation of solar radiation as well as the evaporation calculations, which are not explicitly accounted for in Model 2. This likely led to the reduced model performance exhibitedwhen comparing model simulations to observed data.

**Figure 7.**
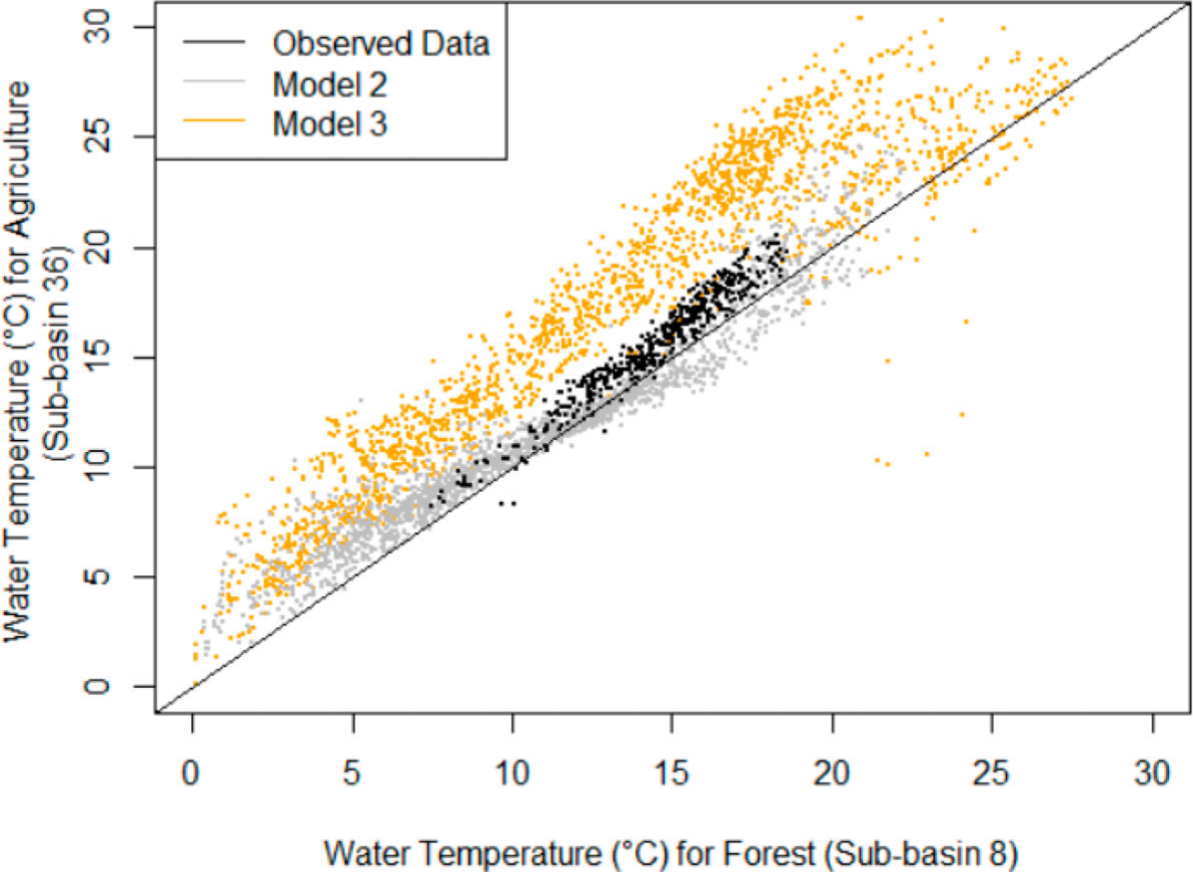
Comparison of observed and simulated stream temperatures for agricultural and forested sub-basins. The observed data (black) show a consistent increase in stream temperatures for the agricultural sub-basin (sub-basin 36). Model 2 does not capture consistent changes in stream temperature associated with land cover type. Model 3 simulates increased temperatures for the agricultural sub-basin, but it overestimates the effect compared to the observed data.

Figure 7 compares these data further by plotting the observed stream temperature as well as simulations using Models 2 and 3 for both the forested (sub-basin 8) and agricultural (sub-basin 36) sub-basins.

The mean (standard deviation) differences between the stream temperature simulations for the forested sub-basin (8) and the agricultural sub-basin (36) were −0.68 °C (1.30 °C) and −4.1 ^◦^C (2.34 °C) for Models 2 and 3, respectively. These simulations can be compared with the difference between the observed data for the forested and agricultural sub-basins (8 and 36): −1.07 °C (0.63 °C). Note that Model 2 gives estimates that are closer to the observed values than Model 3; this is not surprising since the RMSE values for Model 2 were lower than the values for Model 3 (Table 3). However, Model 2 is symmetric about the 1:1 line shown in Figure 7 and does not capture the positive bias of agricultural stream temperatures that is shown by the observed values as well as in the simulated values of Model 3. Model 3 captures the increases in stream temperature associated with the lack of forest cover in the agricultural sub-basin, yet Model 3 overpredicts this effect, and the variation in the simulated values are much greater than Model 2 or the observed data.

Overall, Model 3 may be useful for simulating the watershed-scale impacts of land use conversion from forest to agriculture on stream temperatures; however, model improvement—potentially through improved calibration—is needed to better match observed data. In addition, while Model 3 is able to simulate impacts of land use on stream temperature, this advantage is produced with the trade-off that radiative components exhibit much higher variability, and this noisy fluctuation (which can be seen directly from Figures 5–7) further decreases RMSE values (Table 3).

## 4. Conclusions

This study sought to explicitly incorporate radiative forcing components into an existing mechanistic, semi-distributed stream temperature model Ficklin et al. [10] using SWAT. Ultimately, stream temperature is controlled by different climate components including humidity, wind speed, evaporation, and solar radiation besides air temperature and is also heavily dependent on hydrologic components including surface flow, groundwater, and snow melt processes. Our new model leverages the Ficklin et al. [10] stream temperature model, which accounts for hydrological and climatological components and discretizes the prediction of stream temperature into three parts: (1) Streamflow within a sub-basin, (2) contributing upstream sub-basin hydrologic components, and (3) accounting for the heat exchange between air and water surface, which can be described as a convection term. The extended model replaces the convection K-component term within the Ficklin et al. [10] model with a more comprehensive characterization of radiative energy terms, including solar radiation, atmospheric longwave, land surface longwave, water surface longwave, evaporation, and finally convection drivers. The extended model was used along with the Ficklin et al. [10] model and the linear regression model to simulate stream temperatures within agricultural, forested, and mixed sub-basins within the Marys River watershed. Results showed that all models performed reasonably well, and the Ficklin et al. [10] model outperformed the others. However, the extended model was capable of simulating differences between stream temperatures associated with agricultural and forested watersheds that reflected observed data, although the differences were overestimated. The reduced performance of the extended model that included radiative components might be able to be improved by further calibration; yet, the high variability of the radiative terms is also limiting. For example, the model relies on incoming solar radiation as well as wind velocity, which are difficult to represent over large spatial scales and feature high variability. Alternative formulations of the radiative components should be considered in future work. Bogan et al. [1] suggests using a shading factor instead of a leaf area index, which may lead to more accurate results, assuming shading information is available for the watershed of interest. In addition, alternative estimations for solar radiation, or perhaps any of the radiative energy terms, could improve the model and should be pursued. Overall, incorporating radiative components into the Ficklin et al. [10] stream temperature provides a new mechanism for simulating the effects of alternative land uses on stream temperature within SWAT. This will be especially useful for land managers and decision makers when considering alternative land management scenarios and conservation strategies using SWAT.

## Figures and Tables

**Table 1.** Streamflow calibration parameters.

Parameter	Name	File	Range	Calibration Value
CANMX	Maximum canopy storage (mm H_2_O)	HRU		+25 for FRSE, FRSE
SMFMX	Melt factor for snow on 21 June (mm H_2_O/C-day)	BSN	0–10	8
SMFMN	Melt factor for snow on 12 December (mm H_2_O/C-day)	BSN	0–10	1
LAT_TTIME	Lateral flow travel time (days)	HRU		+5
CH_K2	Effective hydraulic conductivity in main channel alluvium (mm/h)	RTE	0–150	+6
GWQMN	Threshold depth of water in the shallow aquifer required for return flow to occur (mm H_2_O)	GW	0–5000	* 2.5
CN2	Initial SCS runoff curve number for moisture condition II	MGT	0–100	* 0.978
ESCO	Soil evaporation compensation factor	BSN or HRU	0–1	−0.25

* Values are percentages of the original values.

**Table 2.** Basin-wide stream temperature calibration parameters.

Parameter	Name	Range	Calibrated Values
α	Coefficient influencing snowmelt temperature contributions (unitless)	0–1	1.0
β	Coefficient influencing groundwater temperature contributions (unitless)	0–1	0.97
*λ*	Coefficient influencing surface and lateral flow temperature contributions (unitless)	0–1	1.0
*K*	Bulk coefficient of heat transfer (1/h)	0–1	0.025
Lag	Average air temperature lag (days)	0–14	6

**Table 3.** Comparison of root mean square error (RMSE) and percent bias (PBIAS) values for the three tested stream temperature models (daily).

Sub-Basin	Period	RMSE			PBIAS (%)		
		Model 1	Model 2	Model 3	Model 1	Model 2	Model 3
8	2010–2014	3.74	2.18	2.36	23.2	6.9	2.3
15	2010–2014	3.46	1.96	1.96	21.2	3.9	−0.5
17	2010–2014	2.6	1.88	2.72	13.4	−2.6	−8.3
36	2011–2014	2.85	2.28	3.12	13.6	−2.2	−1.0
